# Bilateral Phakomatosis Cesiomarmorata With Ocular Melanocytosis and Secondary Glaucoma

**DOI:** 10.7759/cureus.22861

**Published:** 2022-03-05

**Authors:** Omar Khan, Gorka Sesma, Ahmad Al Jaloud

**Affiliations:** 1 Pediatric Ophthalmology, King Khaled Eye Specialist Hospital, Riyadh, SAU

**Keywords:** oculodermal melanocytosis, childhood glaucoma, nevus of ota, cesiomarmorata, phakomatosis pigmentovascularis

## Abstract

Phakomatosis pigmentovascularis (PPV) is a family of rare congenital diseases where vascular malformation coexists with melanocytic, dermal, or ocular lesions. The cesiomarmorata type is even rarer, and most such cases are reported with unilateral occurrence. We present an atypical case of a patient with bilateral phakomatosis cesiomarmorata, bilateral ocular melanocytosis, and bilateral glaucoma. No malformation to resist aqueous drainage was identified. Long-term management of intraocular pressure (IOP) using topical antiglaucoma medication was successful. This case report refines the clinical presentation of phakomatosis cesiomarmorata and may help diagnose and treat future cases.

## Introduction

Phakomatosis pigmentovascularis (PPV) comprises a family of rare congenital syndromes featuring the coexistence of vascular malformation and dermal or ocular melanocytic lesions. Most cases have a unilateral presentation of skin lesions and ocular involvement [1]. PPV is classified into five distinct types: Type I (capillary malformation, epidermal nevus); Type II (cesioflammea); Type III (spilorosea); Type IV (unclassified, with capillary malformation, dermal melanosis Mongolian spots, nevus of Ota, nevus spilus, nevus anemicus), and Type V (cesiomarmorata) [2], of which cesiomarmorata is less frequent than the other. PPV cesiomarmorata is characterized by the presence of cutis marmorata telangiectasia congenita and cutaneous melanocytosis, including Ota nevus [3]. Women are affected five times more frequently than men [4]. Pathogenesis is associated with somatic mutations in the GNA11 and GNAQ genes [5]. Patients with PPV cesiomarmorata are at risk of developing glaucoma [6].

We discuss the case of a female patient with a rare combination of bilateral PPV cesiomarmorata, bilateral ocular melanocytosis, and bilateral glaucoma diagnosed in early childhood. This case contributes to a refinement of PPV Type V syndrome.

## Case presentation

Early history

An 8-year-old girl with a history of buphthalmos in both eyes and mild photophobia detected since the age of 3 months had been followed up at our hospital, a center for tertiary eye care, since she was 17 months old. The study was approved by the Institutional Review Board of the King Khaled Eye Specialist Hospital (Protocol 21105-CR). The parents signed the informed consent form.

The child was a result of non-consanguineous parents with no family history of glaucoma or similar diseases. She was born with abnormal pigmentation in the sclera of both eyes (nevus of Ota) as well as red-colored patches of skin with widened vessels (cutis marmorata telangiectasia congenita) on both sides of the face, the upper part of the body, and trunk, with all lesions remaining stationary since birth. She was treated for eczema for 16 months with the prolonged application of steroid ointment.

Late-onset bilateral glaucoma

At 17 months of age, she was evaluated under anesthesia. The intraocular pressure (IOP) was 27 mmHg in both eyes, with corneal diameters of 13 mm OD and 12.5 mm OS, pachymetry (520 mm OD and 517 mm OS), and refraction (-0.50 D in OD and +1.00 D in OS). The cupping of the optic nerve was 0.60 in OD and 0.65 on OS. While we could not rule out steroid-induced glaucoma, the first documented diagnosis, at that age, was glaucoma associated with non-acquired systemic disease or syndrome [7], related to PPV. The patient was treated with dorzolamide 2%/timolol 0.5% ophthalmic solution topically administered in both eyes twice a day. The steroid ointments were discontinued immediately following consultation with her dermatologist. After a few months of hypotensive treatment, the IOP was brought under control, and we stopped her antiglaucoma treatment. The patient was medication-free with normal IOP, over the next four years, with a follow up of every six months. However, when she was six years old, her IOP again became high. Her antiglaucoma treatment was reinstated by the glaucoma consultant, this time using brinzolamide 1%/brimonidine tartrate 0.2% fixed combination once a day. Parents were cautioned to administer it with care due to its side effects. The justification for using it was that it had better effectiveness in lowering IOP than topical carbonic anhydrase inhibitors and the possibility of neuroprotection.

The patient's IOP has been stable, lower than 20 mmHg presently, without any negative effects.

Bilaterally symmetric skin lesions

**Figure 1:**
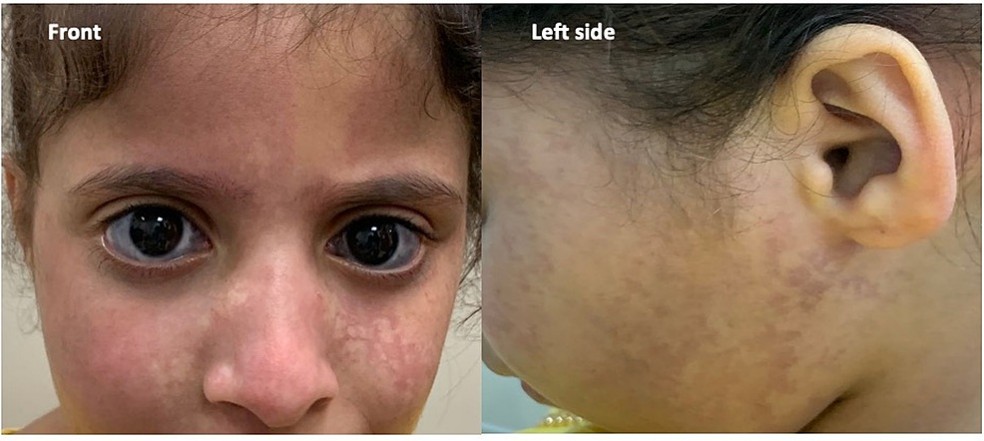
Reticulated macules, scattered along with the three divisions of the trigeminal nerve

Systemic examination revealed erythematous marble-like macules, reticulated with a mottling vascular pattern, aligned with the three divisions of the trigeminal nerve in the bilaterally symmetrical configuration on the face (Figure 1).

**Figure 2:**
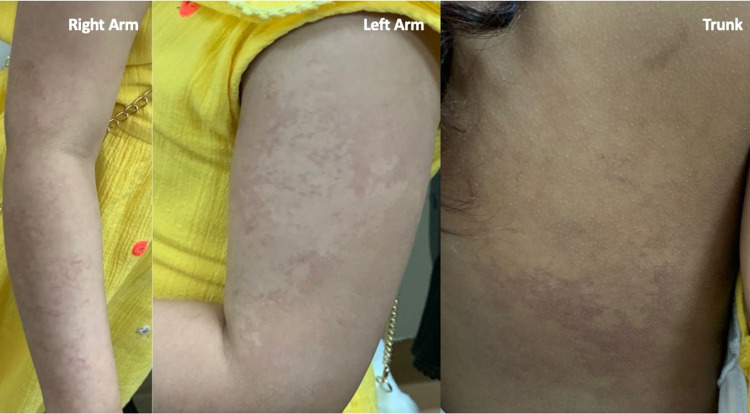
Symmetrical distribution in arms and trunk of marble-like skin aspect

There was hyperpigmented marble-like skin also on the upper limbs and trunk (Figure 2).

There was no asymmetry in the extremities. The patches, persistent after warming, were surrounded by normal, atrophic, or erythematous skin. The observed pattern of lesions is characteristic of cutis marmorata telangiectasia congenita.

Comprehensive eye examination

**Figure 3:**
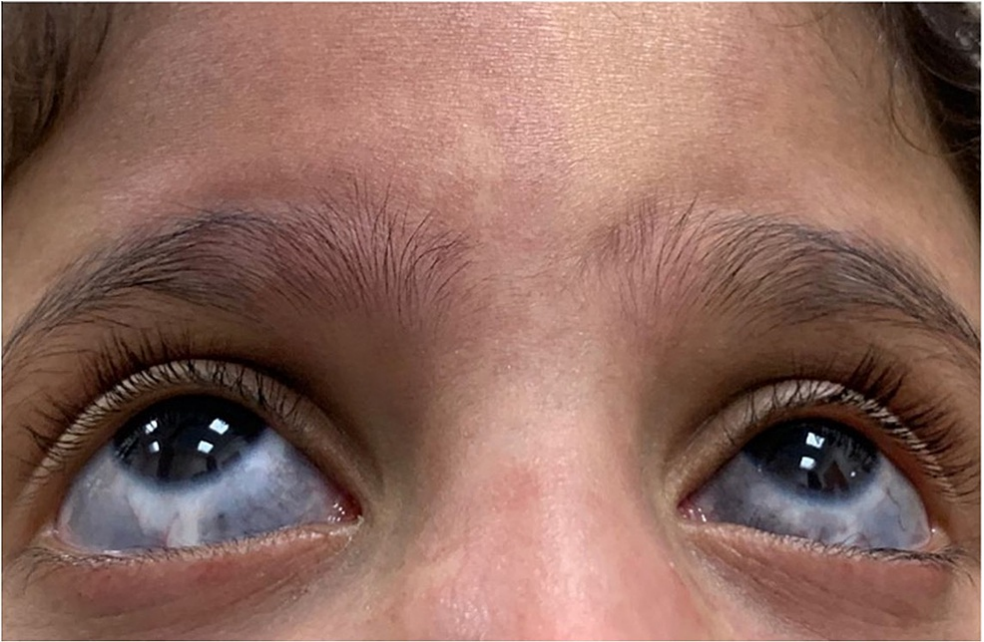
Gray-blue macular hyperpigmentation of the sclera. Nevus of Ota

The visual acuity was 20/25 in both eyes. All the following measurements and inspections were done during a comprehensive ophthalmic examination, performed under topical drops of oxybuprocaine hydrochloride 0.4% (w/v) (Bausch Health Companies Inc., Laval, Canada). The patient was fully cooperative with the examination. The refractive error was −1.5 D in both eyes. The sclera featured hyperpigmentation with melanocytic changes 360° around the cornea in each eye (Figure 3).

**Figure 4:**
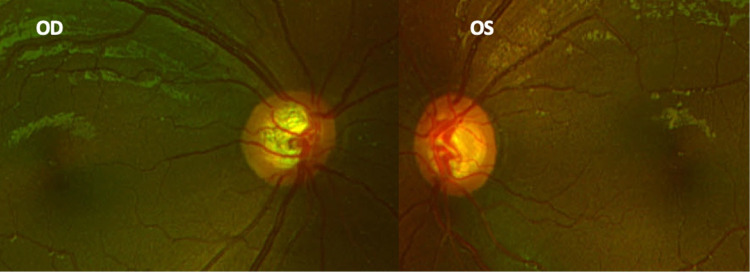
Fundus photograph showing optic nerve pathological cupping in both eyes

The corneas were clear without pigmentation and free of Haab’s striae. The horizontal corneal diameter was 13 mm OD and 12.5 mm OS, but central corneal thickness, obtained by pachymetry, was in the normal range (518 mm OD and 511 mm OS). The anterior chamber was present and deep. The iris showed no signs of alterations, but, as ultrabiomicroscopy revealed, a thickness of 0.83 mm in OD and 0.80 mm in OS. Gonioscopy performed with the Koeppe lens showed four angle elements without pigmentation of the trabecular meshwork, presence of blood in the Schlemm’s canal, and flat iris insertion in both eyes. The lens was clear. IOP, measured with a hand-held tonometer (Tono-pen model AVIA; Reichert Inc., New York, USA), was significantly greater than expected (IOP 25 mmHg OD and 24 mmHg OS). The optic nerve head was pathologically cupped in both eyes (0.75 OD and 0.7 OS), with a notching of an inferior rim in OS and baring of the inferior blood vessel in OD. The dilated fundus examination yielded no other finding (Figure 4).

## Discussion

PPV is a rare congenital syndrome characterized by the simultaneous presence of capillary malformation and pigmentary nevi [8]. It is one of the mosaic heterotrimeric G-protein disorders associated with mutations in GNA11 and GNAQ genes [5]. Nevus of Ota can affect skin, conjunctiva, episclera, iris, or the complete ocular globe. It most typically occurs unilaterally, as reported by Teekhasaenee et al. in a series of studies [9-11]. Rujimethapass et al. described that 91.6% of patients were presented with unilateral involvement of the birthmark [12]. They also determined that the coexistence of nevus of Ota with PPV is predictive of ocular pathology.

Atypical presentation of PPV Type V with bilateral symmetry

Our patient presented with bilateral nevus of Ota. This atypical presentation has a small number of precedents and may have been affected by the presence of nevus of Ota with PPV [13]. Other cases of PPV with ocular involvement have been reported with both bilateral [14] and unilateral presentation [15,16].

The erythematous macular skin lesions in the face and limbs of our patient were indicative of cutis marmorata telangiectasia congenita [17]. However, most cases of congenital cutis marmorata telangiectasia reported in the literature are unilateral and asymmetrical. According to Happle’s classification [2], the combination of this entity with nevus of Ota is known as PPV Type V or phakomatosis cesiomarmorata. It appears to be the least frequent type of PPV, with only seven previous cases mentioned in the literature until 2016 [17], and it is associated with body asymmetry. We did not find asymmetries in the patient’s body, commonly found in this pathology. These patients have an increased risk of developing glaucoma. Rujimethapass et al. found a prevalence of 18% [12]. Multiple studies have demonstrated this association [16,18,19].

Pathophysiology of glaucoma in PPV

A variety of mechanisms can cause glaucoma in PPV. One common mechanism is the increased resistance in the drainage of aqueous humor from the anterior chamber. It can be caused by abnormalities in different components of the anterior chamber anatomy, all of which could lead to IOP elevation [19]. Such abnormalities, including an immature Schlemm's canal structure, narrow anterior angle iris insertion in the ciliary body, and melanocytic infiltration of the trabecular meshwork, were excluded by the comprehensive examination in our patient.

Another mechanism that leads to elevated IOP is through elevated episcleral venous pressure arising from arteriovenous shunting in the presence of an episcleral hemangioma [20], which is a likely cause of late-onset glaucoma. Our findings in the patient support the last option, due to the presence of blood in the Schlemm’s canal in both eyes, although other unknown mechanisms can also be present.

Topical steroids can also cause glaucoma, especially in patients with skin lesions where the rate of absorption of these drugs is increased. Once installed, most of them are refractory to topical antiglaucoma treatment. Our patient responded adequately to antiglaucoma therapy and returned to normal IOP after stopping steroids and kept IOP under 20 mmHg without medications for many years. Although this etiology cannot be ruled out, the case presented did not follow the typical pattern of steroidal glaucoma.

Management of glaucoma in PPV

Patients with PPV need early and regular ophthalmic control, particularly IOP management and preventative vision care. Dermatologists play a vital role in treating the skin problems of these patients and referring them for ophthalmology assessment. Advanced diagnostic imaging techniques, including optic nerve photography, optical coherence tomography, and ultrabiomicroscopy, are valuable for the ophthalmologist to manage these patients. Our patient responded to the first-line therapy of topical antiglaucoma treatment. We believe that this is a reasonable initial step in a conservative approach. However, continued follow-up on this case and further studies involving similar cases are required to establish evidence-based support for its success.​​​​​

## Conclusions

Our patient was diagnosed and treated for bilateral glaucoma associated with the rare Type V PPV in an atypical bilateral presentation of nevus of Ota and cutis marmorata telangiectasia congenita. Comprehensive eye examination helped to rule out malformations to the anterior chamber. The first-line topical medication proved effective in the long-term management of IOP, consistent with earlier case studies on patients with the unilateral presentation of PPV Type V. This case contributes a refinement of the clinical presentation of Type V PPV.
